# Mentalizing in psychotherapeutic processes of patients with eating disorders

**DOI:** 10.3389/fpsyt.2024.1367863

**Published:** 2024-04-19

**Authors:** Almut Zeeck, Inga Lau, Katharina Endorf, Laura Schaefer, Sebastian Euler, Claas Lahmann, Armin Hartmann

**Affiliations:** ^1^ Department of Psychosomatic Medicine und Psychotherapy, Center for Mental Health, Faculty of Medicine, University of Freiburg, Freiburg im Breisgau, Germany; ^2^ Department of Consultation Psychiatry and Psychosomatics, University Hospital Zürich, Zürich, Switzerland

**Keywords:** menatlization based treatment, intervention, in-session, eating disorder, psychotherapy

## Abstract

**Background:**

Improvement in the capacity to mentalize (i.e., reflective functioning/RF) is considered both, an outcome variable as well as a possible change mechanism in psychotherapy. We explored variables related to (in-session) RF in patients with an eating disorder (ED) treated in a pilot study on a Mentalization-Based Treatment (MBT) - oriented day hospital program. The research questions were secondary and focused on the psychotherapeutic process: What average RF does the group of patients show in sessions and does it change over the course of a single session? Are differences found between sections in which ED symptomatology is discussed and those in which it is not? Does RF increase after MBT-type interventions?

**Methods:**

1232 interaction segments from 77 therapy sessions of 19 patients with EDs were rated for RF by reliable raters using the In-Session RF Scale. Additionally, content (ED symptomatology yes/no) and certain MBT interventions were coded. Statistical analysis was performed by mixed models.

**Results:**

Patients showed a rather low RF, which increased on average over the course of a session. If ED symptomatology was discussed, this was associated with significantly lower RF, while MBT-type interventions led to a significant increase in RF.

**Conclusions:**

Results suggest that in-session mentalizing can be stimulated by MBT-typical interventions. RF seems to be more impaired when disorder-specific issues are addressed. Further studies have to show if improving a patient´s ability to mentalize their own symptoms is related to better outcomes.

## 1 Introduction

Mentalizing describes the ability to perceive and understand oneself and others (one’s own behavior/the behavior of others) in relation to inner states, feelings, intentions and desires (1). The capacity to mentalize is important for self-regulation (including the regulation of impulses and affect), as well as the regulation of relationships (1). Therefore, improved mentalizing (operationalized as Reflective Functioning/RF) is discussed both as an desirable outcome of psychotherapy as well as a change mechanism in psychotherapeutic processes (2–4). It was also suggested that a better ability to mentalize is associated with better therapeutic alliances and reduces the risk of treatment drop-out (5, 6). This is an obvious consideration, as a patient who is able to reflect on the mental state of his/her therapist will find it easier not to experience a behavior or intervention as directed against him/herself. To improve mentalizing is the main focus in Mentalization Based Treatment (MBT), an approach originally developed for the treatment of borderline personality disorder – a disorder in which mentalizing is considerably impaired (7, 8). More recently, MBT was adapted for the use in other mental disorders with impairment in mentalizing (9), including eating disorders (10, 11).

RF can be described along different dimensions: It can be related to the self or another person, has a cognitive or affective focus, be implicit or explicit and related to something observable vs. internal mental states (4). Additionally, RF is not only a skill that people have more or less. Mentalizing in a given situation also depends on the context - for example, on the emotional relevance of a given session or the level of arousal induced in the relationship with another individual, including the therapist (7). For instance, high emotional arousal will lead to a fight or flight reaction instead of mentalizing. Therefore, the overall capacity to mentalize a person shows (e.g. in a structured interview like the Adult Attachment Interview), might differ from RF in a specific situation. Such a specific situation are psychotherapy sessions, in which RF is expected to be improved by therapeutic interventions. “In-session” RF (which can be measured with the In-Session-RF-Scale, see below) will depend on the relationship between the patient and the therapist, the topics discussed, the interventions of the therapist and several other factors that might influence the situation (e.g. events prior to the session: if a patient had a conflict with her partner) (12). Furthermore, RF might be impaired concerning the symptoms a patient has. “Symptom specific RF” was defined by Rudden et al. as the ability to reflect on the underlying meaning and affect- or relationship-related function of a symptom (13).

Overall, RF-related process research is in its infancy, although a better understanding of the factors that stimulate mentalizing in sessions and if and how mentalizing is related to productive psychotherapeutic processes is urgently needed. Previous research was able to find a relationship between interventions that are intended to increase RF and higher RF in the respective session (e.g. 14–16). Better RF in a session in turn predicted lower emotional arousal in patients with borderline personality disorder (14). Furthermore, an increase in in-session RF (positive deviation from the individual baseline-level) was shown to be related to less interpersonal problems and a reduction of depressive symptoms in patients with depression and anxiety treated with cognitive-behavior therapy (17).

Eating disorders (EDs) like anorexia nervosa (AN) and bulimia nervosa (BN) primarily affect girls and women in the first half of their lives. AN and BN can easily become chronic with fluctuating courses, and are associated with serious mental and physical consequences (18). Treatment outcomes are not satisfactory, with remission rates barely reaching 50% in adults (19). AN, in particular, carries high mortality rates (20). At the core of psychopathology are difficulties in regulating negative affect (21), along with weight and shape concerns (22). These issues contribute to problematic eating behaviors (restrictive and/or binge eating) and inappropriate compensatory behaviors to prevent weight gain. Maintaining factors include affect intolerance, unfavorable interpersonal interactions, consequences of malnutrition, and habit formation (23). Psychotherapeutic treatment is challenging because of a high ambivalence regarding change (24) and a strong wish for autonomy, while feeling needy and dependent on important others (25). In the majority of studies RF in individuals with ED was found to be impaired, including RF as shown in psychotherapy sessions (26, 27). This is consistent with the fact that problems with the regulation of self-esteem, emotions and impulses on one hand and relationships on the other are at the core of ED psychopathology (18). Therefore, an adapted MBT-approach (MBT-ED) which focuses on an improvement in the capacity to mentalize might be helpful also in the treatment of individuals with an ED. However, there are only few pilot studies evaluating such an approach (11, 28, 29) and one randomized controlled study which included patients with an ED and features of a borderline-personality disorder (30). All of these studies have methodological limitations (observational studies, high drop-out rates) limiting the conclusions which can be drawn from them.

We developed a MBT manual for the treatment of eating disorders (11, 31) and - as a first step - conducted an observational proof-of-concept study in a day hospital setting (11). Results were promising and showed that the program was well accepted by the patients (drop-out rate: 13.2%) and lead to significant reductions in eating pathology (EDE total score) and difficulties with emotion regulation as well as an improvement in RF (11), although overall outcome in ED symptomatology did not differ when compared to a historical matched control group.

The goal of this study, which followed an exploratory approach, is to support a better understanding of processes related to RF in psychotherapy sessions. To this end, we propose to answer the following questions that may inform future research: What is the average RF score of patients during individual MBT-ED sessions? Does RF change over the course of a single session? Are there differences in RF between parts of a therapy session in which eating disorder symptoms are discussed and those in which they are not? Are certain MBT-type interventions associated with increases in RF during the same during the same session sequence? Although the study - due to the few process studies in patients with eating disorders on this topic - was primarily exploratory in nature, we had some expectations based on previous findings. We expected a level of RF below the average values for health individuals. We further expected that MBT-type interventions will be associated with an increase in RF and that RF in average will increase over the course of a session (as we analyzed MBT-oriented sessions with corresponding objectives).

## 2 Method

### 2.1 Study design – original study

The original “proof-of-concept”-study was prospective and observational. It was approved by the local ethics committee (No 448/17) and conducted in a day hospital, which provides an MBT-ED program for six patients with an ED at a time. All consecutively admitted patients with an ED over a period of 2 years were asked to take part in the study. In this time period, 38 out of 40 ED-patients admitted could be included. Inclusion criteria were a diagnosis of anorexia nervosa (AN), bulimia nervosa (BN) or other specified feeding and eating disorders (OSFED) according to DSM-5 (mental diagnoses were given after a SCID-5 interview), age ≥ 18, BMI ≥ 14.5 kg/m² and an indication for day hospital treatment (11). Exclusion criteria were psychoses, substance dependency, bipolar disorder, organic brain disease, dementia, severe somatic illness or acute suicidal ideation. The multimodal treatment program includes two MBT individual sessions per week (50 min, 25 min) and a one-weekly MBT-group therapy session besides further components [e.g. art and body therapy, work with an eating diary; for details see (11)]. Therapists were trained in MBT and supervised by a certified MBT supervisor. Individual sessions were videotyped and assessed for MBT adherence which included feed back to the therapist after every 4th session. Main time points of assessment were admission, discharge and follow up assessments three and twelve months after discharge.

### 2.2 Process study

Every second patient was asked to take part in a process study (not every patient could be included due to the high effort involved). To study psychotherapeutic processes, we focused on individual treatment sessions. The second session and every forth of following sessions were included and transcribed according to the rules of Mergenthaler (32). Session transcripts were divided into 3-minute sequences. Thus, a therapy session of about 50 minutes yields 17 coded segments, with a time variable ranging from 3 to 51 by 3. Each sequence of the included sessions was rated for RF using the In-Session-Reflective Functioning-Scale (12). The scale ranges from -1 (refusing to use RF) and 0 (no RF) to values between 1 and 9 (1-4 low RF, 5 = normal RF, 6-9 high RF). The ratings were conducted by two trained and reliable raters (ICC = .81 (27);). In addition to RF, the content of a sequence was coded. It was coded in terms of a focus on eating symptomatology (1 = yes/defined as sequences with a focus on ED symptoms vs. 0 = no/sequences without this focus) and if two types of MBT- interventions were used in the respective time segment: „demand”-interventions (prompting a patient to reflect on or explore a topic in more detail) and empathic validation (actively validating the emotional experience reported by a patient) (1 = yes/sequences with MBT intervention; 0 = no/sequences without MBT intervention).

We decided to exclude the last six minutes of each session from the analysis, because of typically very low RF (tested with mixed model: -0.64 RF compared to the other time segments; p < 0.0001), potentially changing the trajectory to non-linear. We considered the last minutes (talking out/saying goodbye, appointments, organizational issues) therefore as not representative of the psychotherapy process and the capacity of a patient to mentalize.

### 2.3 Psychometric measures

Eating psychopathology was measured with the Eating Disorder Examination Interview (EDE) interview (33, 34) and the Eating Disorder Inventory self-report questionnaire (EDI-2) (35, 36), general psychopathology with the Symptom-Check-List (SCL-90-R) (37), see also (11). In the original study, time points of measurement were admission, discharge as well as three and twelve month after discharge.

### 2.4 Statistical analysis

In order to account for the hierarchical structure of the data, we used mixed models to estimate linear trends of RF within sessions and it’s relations to session process. The analyses were computed with R (V4.2.2) and the package lme4 (V.1.1-32; Syntax see **Table 1**; REML estimation).

**Table 1:** Results of mixed model.

Random Effects	Groups		*Variance*	*SD*	*corr*
	PatID: SessionID		0.902	0.950	
	Minute		0.00036	0.019	-.40
	Residual		0.782	0.884	
Fixed Effects	*Estimate*	*SE*	*df*	*t*	*p <*
Intercept	3.244	0.133	104.8	24.445	0.0001
ED-Focus	-0.2021	0.074	1058	-2.725	0.0065
Demand	0.2893	0.063	1038	4.641	0.0001
Empathic Validation	0.2580	0.109	1044	2.361	0.0184
Minute (RF/min)	0.0079	0.003	69.15	2.623	0.0107

Intercept = Starting level of RF; SE, standard error; SD, standard deviation; corr: correlation.

R Syntax (package lme4, REML estimation): Model <- lmer(RF ~ 1 + ED-Focus + Demand + EmpVal + Minute (Minute | PatID : SessID), data=dt1).

## 3 Results

19 patients were included in the study. 77 sessions and 1232 session sequences were available for the analysis. For a sample description see **Table 2**.

**Table 2:** Sample description.

Variable (*N* = 19)		M (SD) / N (%)
Age [years]		29,8 (11,7)
Female		19 (100%)
Education	< 12 years ≥12 years	6 (31,6%) 13 (68,4%)
Duration of illness (years)		9.8 (11.8)
Admission BMI [kg/m²]		20.0 (5.5)
	< 17.5 kg/m²	8 (42.1%)
	≥17.5 kg/m²	11 (57.9%)
ED diagnosis	AN AN partially remitted BN OSFED	8 (42.1%): 6 restrictive, 2 binge-purge 1 (5.3%) 8 (42.1%) 2 (10.5%)
EDE^#^ (T-score) EDI-2^##^ (T-scores)	Total score Drive for thinness Bulimia Body dissatisfaction	81.9 (36.5) 77.4 (21.5) 75.6 (26.1) 65.9 (13.6)
SCL-90 (GSI)*		1.23 (0.53)
Day hospital treatment	Duration (weeks)	12.97 (4.92)

# EDE, Eating Disorder Examination Interview (34); ## EDI-2, Eating Disorder Inventory (36); * SCL-90 (GSI), Global Severity Index of the Symptom Check List (37).

**Figure 1:**
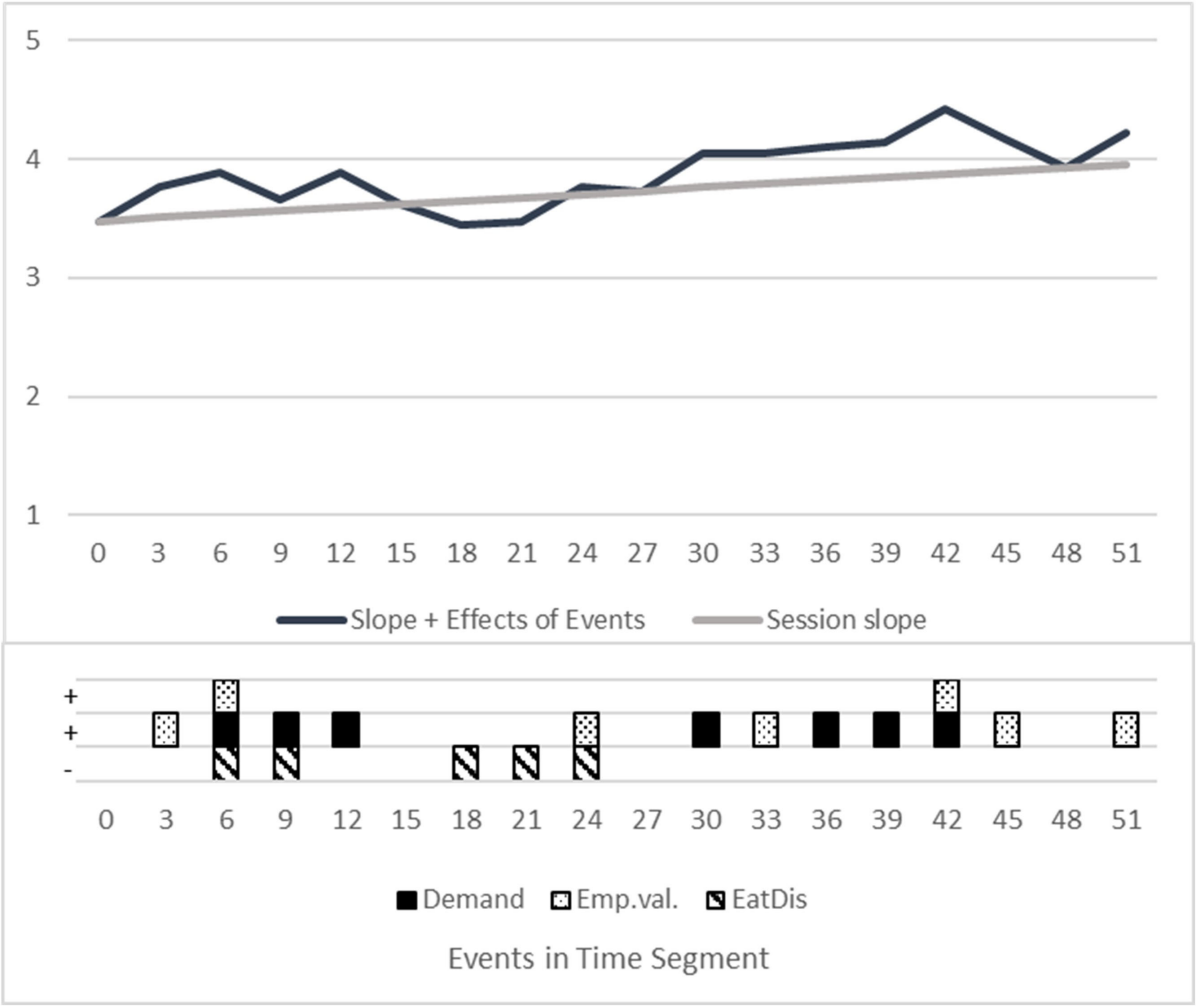
Visualization of a constructed therapy session. Constructed trajectory, showing the estimated impact of interventions on RF with a hypothetical pattern of interventions and ED focus. “Session slope”: RF Mean session trajectory with intercept = 3.48 RF and estimated increase of 0.48 RF (from minute 1 to minute 50). “Events”: Estimation of fixed effects directly in the segment of occurrence. Slope + Effects of Events: Mean course PLUS effects of all events / interventions. X-Axis/Time: Divided into the rated segments of 3 minutes. Squares with patterns: Constructed occurrences of interventions, coded yes=1, no = 0. Random Effects: Not shown, as this is a constructed single case. Random intercepts and slopes differ individually.

Overall, patients showed a low level of RF in sessions (M = 3.48). It did not differ between patients with a BMI below 18.5 kg/m² (M = 3.54; N = 9) and those with a BMI of 18.5-25 kg/m² (M = 3.47; N = 8). Two patients with a BMI > 25 kg/m² had a lower RF (M = 2.50; N = 2). On average, RF increased over the course of a session (Intercept = 3.24, slope = +0.0079/min = +0.48/50min), see **Table 1**. Talking about eating-disorder related themes was associated with significantly lower RF (-0.20) within the respective, 3-minute long sequences of the sessions. Demand-interventions were positively associated with higher RF (+ 0.29) within the respective 3-minute sequence, this also applied to empathic validation (+ 0.26). **Table 1** shows the formula and the estimates of the mixed model. For an illustration and better understanding, a constructed trajectory of a singe case is visualized in **Figure 1**.

## 4 Discussion

The average RF shown in the sessions was low (38, 39). This is consistent with preliminary findings in patients with EDs (10, 26). It has to be taken into account that we assessed in-session RF, which depends on the process in each session and interventions used by the therapist. However, if we understand a psychotherapeutic session as a situation in which RF is usually challenged, average in-session RF will be an indicator for the overall capacity to mentalize (17). Talia et al. (12) found a moderate correlation between In-Session-RF and RF as assessed with the Adult Attachment Interview (AAI), probably due to the less standardized situation in therapy sessions (the AAI is a structured interview that uses so-called “demand” questions to stimulate RF). Nevertheless, patients with higher RF ratings in the AAI, showed also a better capacity to mentalize in psychotherapy sessions.

We found that RF increased over the course of a session. This might reflect a process of increasing reflection in this session, which would be intended in an MBT-oriented treatment (7, 40). However, we cannot rule out that the finding is unspecific and for example due to the typical structure of a psychotherapy session: At the beginning the focus is on getting into contact and establishing a safe atmosphere, before more challenging topics are discussed. However, despite the general increase in RF, there could be fluctuations in RF that depend, for example, on the extent to which a patient feels perceived by their therapist and considers their interventions to be credible and trustworthy (41, 42).

In terms of content, RF was lower in transcript sequences in which symptomatology was discussed. This could mean that mentalizing might „break in” when disorder-specific topics are addressed and be interpreted as a reduced capacity to reflect on the function and meaning of symptoms. It is an important question, if this correlation changes over the course of a successful treatment (that psychotherapy leads to an increase in RF in the context of eating-disorder related themes) and if such an improvement in symptom-related RF is finally related to outcome. This would need to be investigated in a larger prospective study in the future. As mentioned in the introduction, symptom-specific RF was previously shown to be relevant for change: A study on patients with panic disorder, the Cornell-Penn-Study, found that an increase in panic-specific RF in cognitive-behavioral as well as psychodynamic psychotherapy mediated a better treatment outcome (43, 44).

Finally, we found that sequences with demand interventions or empathic validation showed increased mentalizing in the patient. Although we did not study the time sequence (if patients mentalized directly following these interventions), the finding suggest that both interventions might simulate RF. This would be a replication of previous findings, where could be shown that that MBT-type interventions in cognitive-behavioral and psychodynamic treatments of AN were associated with an increase in in-session RF (15). Interestingly, both interventions are correlated with a similar increase in RF, although they differ in terms of their aim and might work through different mechanisms: While demand-interventions intend to directly stimulate RF, empathic validation is used to give the patient a feeling of being understood and intends to validate his experience emotionally. This is considered to be a necessary base for mentalizing, especially in situations, in which a patient is emotionally challenged (42).

The study has several limitations, which include the small sample size (which did not allow to analyze for influences of weight status) and the heterogeneous group of patients with an ED. An *a priori* power analysis was not conducted, power and sample size depended on the design of the primary study. Exploratory data analyses revealed no consistent pattern of non-linear trajectories. Therefore, we decided to model linear trajectories only. The sample consisted of women only. There is no baseline assessment of RF, e. g. with the Adult Attachment Interview and the RF-Rating-Scale, measuring by overall capacity of the patients to mentalize. Interventions like “demand” and “empathic validation” could be considered rather “unspecific” interventions without the context of the situation in which they are used and we did not assess a lot of other therapeutic interventions that might or might not contribute to RF.

In summary, we were able to show that RF in psychotherapy sessions with patients with an ED is not only context-dependent, but also depends on the content discussed. The ability to mentalize appears to be particularly impaired when disorder-specific topics (relating to food, body and weight) are addressed. Future studies should answer the question of whether a therapeutic focus on mentalizing eating disorder-specific experiences and beliefs during a session and an improvement in symptom-specific RF is a significant mediator of treatment success.

## Data availability statement

The raw data supporting the conclusions of this article will be made available by the authors, without undue reservation.

## Ethics statement

The studies involving humans were approved by the local ethics committee of the University of Freiburg, No 448/17. The studies were conducted in accordance with the local legislation and institutional requirements. The participants provided their written informed consent to participate in this study.

## Author contributions

AZ: Conceptualization, Funding acquisition, Investigation, Methodology, Project administration, Resources, Supervision, Writing – original draft, Writing – review & editing. IL: Data curation, Investigation, Project administration, Writing – review & editing. KE: Data curation, Investigation, Writing – review & editing. LS: Resources, Writing – review & editing. SE: Supervision, Writing – review & editing. CL: Writing – review & editing. AH: Conceptualization, Data curation, Formal Analysis, Methodology, Writing – review & editing.
